# The use and preference of artemether as a first-choice treatment for malaria: results from a cross-sectional survey in the Bata district, Equatorial Guinea

**DOI:** 10.1186/s12936-018-2254-0

**Published:** 2018-03-09

**Authors:** Maria Romay-Barja, Policarpo Ncogo, Gloria Nseng, Maria A. Santana-Morales, Pedro Berzosa, Zaida Herrador, Basilio Valladares, Matilde Riloha, Agustin Benito

**Affiliations:** 1 Centro Nacional de Medicina Tropical, Instituto de Salud Carlos III, Madrid, Spain; 2 Red de Investigación Colaborativa en Enfermedades Tropicales, RICET, Madrid, Spain; 3 Centro de Referencia de Control de Endemias, Malabo, Equatorial Guinea; 4 Ministerio de Salud y Bienestar Social, Malabo, Equatorial Guinea; 5 Instituto Universitario de Enfermedades Tropicales y Salud Pública de Canarias, Universidad de La Laguna, Tenerife, Spain

**Keywords:** Malaria, Treatment, Artemether, Artemisinin-based combination therapy, ACT, Practitioners, Behaviour

## Abstract

**Background:**

Malaria is endemic in Equatorial Guinea with stable transmission, and it remains a major cause of morbidity and mortality in children under 5 years of age. Adherence to artemisinin-based combination therapy (ACT) as a first-line treatment for uncomplicated malaria is critical to malaria control. Six years after the introduction of artesunate-amodiaquine (AS/AQ) therapy in Equatorial Guinea, adherence to the first-line treatment seems to be low in the Bata district. The factors associated with the choice of malaria treatment have not been studied previously in this area; therefore, this study aimed to analyse the preference and use of artemether as malaria treatment and its related factors in the Bata district of Equatorial Guinea.

**Methods:**

In 2013, a cross-sectional study was conducted in the Bata district, which involved 428 households. Bivariate and multivariate statistical analyses were conducted to determine the relevance of socio-economic, geographical, and behavioural factors that played a role in the preference and use of artemether as malaria treatment.

**Results:**

Artemether was considered the best treatment for malaria by 110 caregivers (26%), and was the antimalarial most administrated in the Bata district. It was prescribed to 117 children (27.34%); while, only 6.78% were administered AS/AQ. Caregivers living ≤ 3 km from the nearest health facility were almost two times more likely to consider artemether as the best treatment than those living farther away (95% CI 0.31–0.86). Caregivers with at least a secondary school education were 2.7 times more likely to consider artemether as the best treatment than those less educated. Children whose caregivers considered artemether the best treatment against malaria were five times more likely to be treated with artemether than children with caregivers who did not consider it the best (OR 5.07, 95% CI 2.93–8.78). In contrast, children that reported weakness as a symptom were less likely to be treated with artemether than those with other symptoms (OR 0.47, 95% CI 0.28–0.78).

**Conclusion:**

Caregivers, public and private health staff, and drug sellers need to understand the importance of using ACT to treat uncomplicated malaria and the dangers of using artemisinin monotherapy.

## Background

There are 216 million cases of malaria and more than 445,000 related deaths per year. Most of these deaths occur in sub-Saharan Africa, with 97% of deaths occurring in children aged less than 5 years due to *Plasmodium falciparum* malaria [1].

Malaria case management, based on an early diagnosis and prompt treatment with effective anti-malarial drugs, remains the major strategy for effective control of malaria. However, poor perceptions about malaria and poor malaria drug treatment practices have contributed to the emergence of widespread *P. falciparum* resistance [2]. After proving the resistance of *Plasmodium* to chloroquine, the World Health Organization (WHO) recommended the use of an artemisinin-based combination therapy (ACT) as the first-line treatment for uncomplicated malaria in 2001 [3]. Unfortunately, emergence and spread of artemisinin resistance has been detected in Southeast Asia [4] and is now threatening global malaria control and elimination efforts, especially in sub-Saharan Africa, where the disease burden is highest and systems for monitoring treatment compliance and containment of resistance are weak [5]. Adherence to this ACT first-line treatment is critical to the success of malaria control.

Despite progress made reinforcing access to treatment, WHO reported that only 30% of diagnosed children less than 5 years old received an anti-malarial drug during the last year, and the proportion receiving ACT was only 14% [1]. Many years after the introduction of ACT, poor access to treatment; inappropriate treatment practices such as presumptive treatment, co-medication, use of low quality ACT, artemisinin monotherapy and the expensive price of ACT persist [6]. Low use of ACT may increase the malaria burden and the risk of widespread parasite resistance and lead to treatment failure [7].

Malaria has stable transmission and is endemic in Equatorial Guinea [8] and remains a major cause of morbidity and mortality of children under 5 years of age [9]. A malaria control programme was introduced in the mainland region in 2007, under the Equatorial Guinea Malaria Control Initiative (EGMCI). Case management was improved with the distribution of free ACT to all health facilities and Community Health Workers (CHWs). The initiative was largely funded by The Global Fund to Fight AIDS, Tuberculosis, and Malaria (GFATM), and it was implemented by the government of Equatorial Guinea, in collaboration with several international organizations. The first and second line treatments for uncomplicated malaria in Equatorial Guinea are artesunate-amodiaquine (AS/AQ) and artemether/lumefantrine (AL), respectively. According to the Malaria National Treatment Guide, intravenous artesunate injection is recommended for the management of severe malaria. If artesunate is not available, quinine and artemether should be administered until the patient recovers consciousness, then full AS/AQ therapy should be administrated [10]. Unfortunately, with the withdrawal of the GFATM funding in 2011, the EGMCI stopped its main activities in mainland Equatorial Guinea. Universal access to ACT has not been assured ever since [11] and no actions have been implemented to improve ACT adherence in the district of Bata. Equatorial Guinea is one of the African countries with the lowest percentage of malaria cases treated with antimalarial medicines [12]. In 2015, WHO estimated there were about 310,000 malaria cases in Equatorial Guinea, but only 40,911 ACT delivered [1]. The use of oral artemisinin monotherapy was forbidden in Equatorial Guinea in 2014 [10].

Six years after the introduction of AS/AQ therapy in Equatorial Guinea, adherence to the first-line treatment seems to be non-existent in the Bata district where there is evidence of an alarming use and preference for artemether monotherapy as the first choice for malaria treatment. The Bata population considers artemether the best treatment for malaria, especially in urban areas [13] and, according to caregiver reports, artemether is the anti-malarial most often administrated to children, regardless of where they seek treatment [14]. The use of artemisinin monotherapy involves serious risks because adherence to these relatively long treatment regimens is low, leading to parasite resistance and illness recrudescence [15]. Detailed knowledge about the use and preference of artemether as first choice treatment is needed in order to design effective actions addressed to protect the therapeutic life of ACT in Bata district.

Understanding the treatment behaviour and use of anti-malarial drugs is crucial to malaria control and elimination [16]. Household decisions about which drugs to use are usually based on prior illness and treatment experiences, local beliefs, understandings of illness aetiologies, recognition of symptoms, influence of social networks, perceived effectiveness of a treatment, and available options [17]. Several factors may be responsible for a household’s non-adherence to first-line malaria treatment, including the level of education of the caregiver, employment, children’s ages and symptoms, the perception of the severity of the disease, time lapse during the search for treatment, and the source of care [18, 19].

Further research on the use and choice of malaria treatments and its determinants is necessary to strengthen interventions, improve malaria case management, and avoid the emergence and spread of artemisinin resistance. This study aimed to analyse the preference and use of artemether as malaria treatment and its related factors in the Bata district of Equatorial Guinea.

## Methods

### Study area and population

Bata is the largest district of Equatorial Guinea, with a population of 244,264 inhabitants [17]. Despite the efforts made by the EGMCI, the malaria prevalence has remained high (41.2%) in children under 15 years old [20]. The District’s public health facilities comprise a network of ten health centres, two rural and eight urban, and one regional hospital located in the city of Bata. There are also private health facilities in the Bata district, including two hospitals and about 23 clinics, all in the urban area of Bata city [21]. About 156 pharmacies and other drug sellers are located throughout the rural and urban areas of the District. In rural areas, where the majority of the population lives > 3 km from a health facility, CHWs were trained to manage the malaria diagnosis and treatment [13].

### Survey design and data collection

This cross-sectional study was implemented in June–August 2013 in the Bata district of mainland Equatorial Guinea. This survey was part of a project that aimed to provide baseline data on the prevalence of malaria, the genetic characteristics of *P. falciparum*, vectors involved in the transmission, and to provide information about malaria-related knowledge, attitudes, and practices of the target population.

A multistage, stratified cluster sampling strategy was used. Primary sampling units were rural villages and urban neighbourhoods, which were randomly selected with a probability proportional to size to improve the accuracy of the sample. Then, 440 households from each cluster were randomly selected from an updated census provided by the head of the village or neighbourhood.

Household caregivers were identified in each house with at least one child less than 15 years of age, and they were asked about their knowledge, attitudes, and treatment-seeking behaviour related to the most recent malaria episode in a child under their care. An open-ended questionnaire was administered by trained field workers. Information regarding household social characteristics was also recorded. The questionnaire was previously tested and translated into the main local language, Fang. Caregivers could be interviewed in Spanish or Fang, both official languages in the country. The details of the survey have been described previously [20, 21].

### Data analysis

A wealth indicator variable that served as a proxy for socio-economic status was created with household-owned assets, housing characteristics, and the type of access to water and sanitation using principal component analysis [22, 23]. The first principal component was considered the summary measure of socio-economic status, and subsequently divided into quintiles to assign households to different wealth strata. A malaria knowledge score was created to categorize respondents with poor or good knowledge, including the caregiver’s knowledge about malarial transmission, disease symptoms, prevention, treatment for children, and treatment centres. Good or poor knowledge of malaria disease symptoms, transmission, and prevention was defined as scores above or within and below the overall median, respectively [13].

Frequencies and percentages were used to summarize data and to assess factors related to the use of artemether as treatment and considering artemether the best treatment. The mean and standard deviation or median and interquartile range were calculated for continuous variables that were or were not normally distributed, respectively. Student’s t test and χ2 tests were performed for continuous and categorical variables, respectively. Comparisons with p values < 0.05 were considered significant. Bivariate analyses of the associations between the independent variables and artemether, as the treatment received and as the malaria treatment preferred, were conducted using simple logistic regression. Independent variables that were significantly associated at the p < 0.10 level were included in the multivariable analysis. The collinearity between independent variables was checked and when present, the variable explaining less data distribution was removed. Logistic regression models were obtained using a manual backward stepwise procedure. The adjusted odds ratio (aOR) and 95% confidence interval (95% CI) were computed; p values ≤ 0.05 were considered statistically significant. The sampling design was considered in the analysis. Data analysis was performed using STATA software version 12.

### Ethical approval

This study was approved by the Ministry of Health and Social Welfare of Equatorial Guinea and the Ethics Committee of the Spanish National Health Institute, Carlos III (CEI PI 22_2013-v3). Written informed consent for participation in the study was obtained from the caregivers interviewed and from the heads of the households.

## Results

### Descriptive statistics

**Fig. 1:**
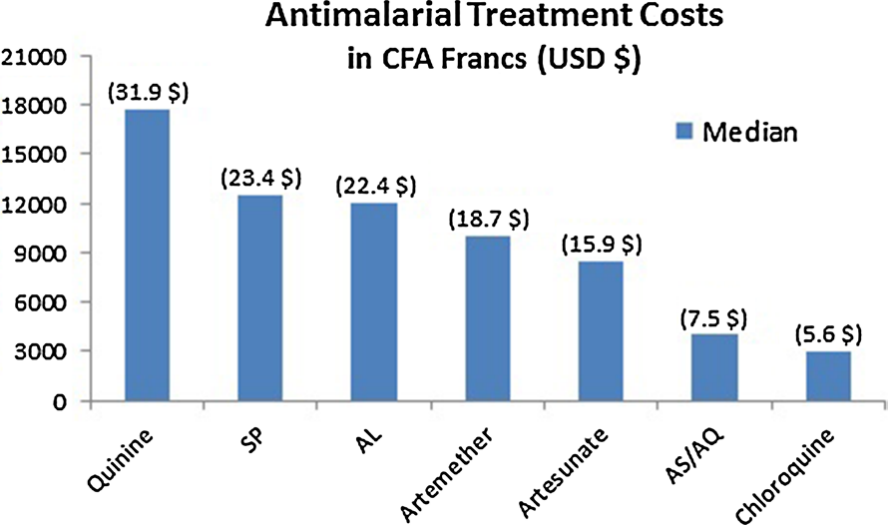
Malaria treatment costs reported by caregivers according to the treatment prescribed in the Bata district. *SP* sulfadoxine pyrimethamine, *AL* artemether-lumefantrine, *AS/AQ* artesunate-amodiaquine

A total of 428 caregivers were asked what they considered the best treatment for malaria. Artemether was the therapy most mentioned, by 110 caregivers (25.7%); while, AS/AQ was only mentioned by 6.5% of caregivers. When asked about the treatment prescribed to their child during the last malaria episode, the anti-malarial drugs most administrated were artemether to 117 children (27.3%) and sulfadoxine/pyrimethamine (SP) to 59 (13.8%); while, AS/AQ was only administrated to 6.8% of the children. Treatment cost was also reported. Treatment with artemether was more expensive than AS/AQ (Fig. 1), with a median costs of 10,000 (IQR:6700–18,000) CFA (18.7 USD) and 4000 (IQR:2000–15,750) CFA (7.5 USD), respectively.

### Considered artemether as the best treatment

Most of the caregivers that considered artemether the best treatment for malaria (Table 1), were aged between 25 and 34 years (45.5%), had attended at least secondary school (75.5%), had a good Malaria Knowledge Score (60.0%) and around 74.5% of caregivers lived in urban areas ≤ 3 km from the nearest health facility. When asked about why they consider artemether the best drug to treat malaria, 60.9% answered that “it is the treatment that makes them feel better” (while other treatments have side-effects) and 20.9% said that “it is the treatment they always give to us”. Almost half of the caregivers (43.6%), who considered artemether the best treatment, received advice from a hospital or health centre (66.7%) before treating the last malaria episode in a child under their care but differences with those that did not received advise not reach significance.

**Table 1:** Household characteristics related to the preference for artemether as malaria treatment

	Considered artemether the best malaria treatment				p value
	No		Yes		
	n	%	n	%	
Caregiver age (years)					
15–24	65	20.4	21	19.1	
25–34	103	32.4	50	45.5	
35–44	61	19.2	21	19.1	
45–54	56	17.6	15	13.6	
≥ 55	33	10.4	3	2.7	0.034
Education					
Primary school or less	174	54.7	27	24.5	
At least secondary school	144	45.3	83	75.5	0.000
Malaria knowledge					
Poor	206	64.8	44	40.0	
Good	112	35.2	66	60.0	0.000
Area					
Rural	145	45.6	28	25.5	
Urban	173	54.4	82	74.5	0.000
Distance to nearest health facility					
≤ 3 km	166	52.2	83	75.5	
> 3 km	152	47.8	27	24.5	0.000
Wealth quintiles					
Poorest	72	22.6	15	13.6	
Second	59	18.6	31	28.2	
Middle	64	20.1	16	14.5	
Fourth	64	20.1	23	20.9	
Richest	52	16.4	32	29.1	0.026
Delay in seeking treatment					
≤ 24 h	124	39.0	49	44.5	
> 24 h	114	35.8	34	30.9	0.275
Have you received advice on treating malaria?					
No	230	72.3	62	56.4	
Yes	88	27.7	48	43.6	0.002
To have a malaria case in the house					
No	90	28.3	36	32.7	
Yes	201	63.2	71	64.5	0.605
Sex of child	318		110		
Male	158	49.7	64	58.2	
Female	160	50.3	46	41.8	0.124
Age group (years)					
< 1	40	12.6	21	19.1	
1–5	191	60.1	60	54.5	
> 5	87	27.4	29	26.4	0.236

### Received artemether as treatment

Most of the 117 children treated with artemether were aged between 1 and 5 years (64.1%) with almost no difference in sex, most lived in urban areas (65.0%), at ≤ 3 km from the nearest health facility (66.7%). Regarding the caregivers, 65.8% were aged less than 34 years, 66.7% had finished at least secondary school, and 57.3% had good Malaria Knowledge Score. Almost half of the caregivers (49.6%) whose child received artemether considered it the best treatment for malaria and only 7.7% considered AS/AQ the best. Most of the children treated with artemether were brought to a hospital to seek care (63.2%), but there was not a statistically significant difference when compared with the other sources of care. Almost 60.0% of the children treated with artemether sought treatment during the first 24 h after the onset of symptoms (Table 2).

**Table 2:** Characteristics of children treated with artemether and their households in the Bata district

	Artemether				p value
	No		Yes		
	n	%	n	%	
Age group (years)					
< 1	49	15.8	12	10.3	
1–5	176	56.6	75	64.1	
> 5	86	27.7	30	25.6	0.253
Sex of child					
Male	155	49.8	67	57.3	
Female	156	50.2	50	42.7	0.171
Area					
Rural	132	42.4	41	35.0	
Urban	179	57.6	76	65.0	0.165
Distance to nearest health facility					
≤ 3 km	171	55.0	78	66.7	
> 3 km	140	45.0	39	33.3	0.029
Wealth quintiles					
Poorest	72	23.2	15	12.8	
Second	59	19.0	31	26.5	
Middle	64	20.6	16	13.7	
Fourth	64	20.6	23	19.7	
Richest	52	16.7	32	27.4	0.008
To have a malaria case in the house			195		
No	90	28.9	36	30.8	
Yes	194	62.4	78	66.7	0.983
Caregiver characteristics					
Caregiver age					
15–24	62	19.9	24	20.5	
25–34	100	32.2	53	45.3	
35–44	58	18.6	24	20.5	
45–54	58	18.6	13	11.1	
≥ 55	33	10.6	3	2.6	0.008
Education					
Primary School or less	162	52.1	39	33.3	
At least secondary school	149	47.9	78	66.7	0.001
Malaria knowledge					
Poor	200	64.3	50	42.7	
Good	111	35.7	67	57.3	0.000
Best treatment					
Artemether	52	16.7	58	49.6	0.000
AS/AQ	19	6.1	9	7.7	0.555
Source of treatment					
Pharmacy	26	8.4	13	11.1	
Traditional healer	1	0.3	0	0.0	
Private doctor	44	14.1	14	12.0	
Health centre	52	16.7	15	12.8	
Hospital	123	39.5	74	63.2	
CHW	2	0.6	0	0.0	
Neighbour	1	0.3	0	0.0	0.188
Delay					
≤ 24 h	120	51.9	53	58.9	
> 24 h	111	48.1	37	41.1	0.262

**Fig. 2:**
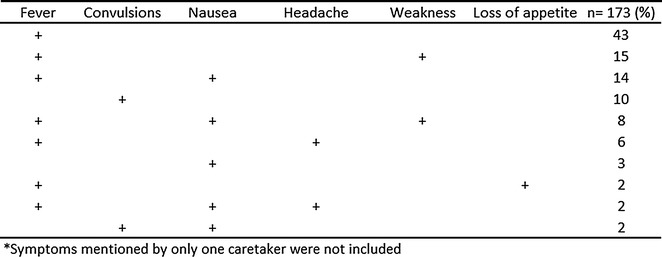
Symptoms of children treated with artemether

The three malaria symptoms most mentioned by the caregivers of children that received artemether were fever (84.6%), nausea (26.5%), and weakness (23.9%). Nausea and weakness were the only two symptoms significantly associated with receiving artemether treatment. The signs and symptoms mentioned by caretakers of children treated with artemether are presented in Fig. 2.

### Factors related to the treatment preferences

Once adjusted for other variables, only the educational degree, Malaria Knowledge Score, and the distance to the health facility remained significant. Household caregivers that lived ≤ 3 km from the nearest health facility were 1.9 times more likely to consider artemether the best treatment for a child with malaria than those living farther away after adjusting for other variables (95% CI 0.31–0.86). Also, caregivers with at least a secondary school education who had good malaria knowledge score were 2.7 and 1.9 times more likely to consider artemether the best treatment than those with less education and malaria knowledge, respectively (Table 3).

**Table 3:** Factors associated with the preference for artemether as treatment by multiple logistic regression

	Unadjusted OR (95% CI)	Adjusted OR (95% CI)^a^
Area		
Rural		
Urban	2.45 (1.50–4.01)	
Distance to nearest health facility		
≤ 3 km		
> 3 km	0.36 (0.22–0.58)	0.52 (0.31–0.86)
Wealth quintiles		
Poorest		
Second	2.15 (0.98–4.69)	
Middle	2.22 (1.00–4.95)	
Fourth	2.38 (1.09–5.21)	
Richest	3.47 (1.59–7.60)	
Caregiver age (years)		
15–24		
25–34	1.50 (0.82–2.74)	
35–44	1.07 (0.53–2.15)	
45–54	0.83 (0.39–1.77)	
≥ 55	0.28 (0.08–1.04)	
Education		
Primary school or less		
Secondary school or more	3.71 (2.24–6.16)	2.74 (1.74–4.30)
Malaria knowledge		
Poor		
Good	2.76 (1.75–4.36)	1.99 (1.27–3.12)
Did you receive advice to treat malaria?		
No		
Yes	2.02 (1.28–3.19)	

^a^Only for the variables that stay in the model

### Factors related to being treated with artemether

Children with weakness were 2.1 less likely to have received artemether, after adjustment for other variables; while, children whose caregivers had good malaria knowledge score were 1.8 times more likely to have received artemether than children with caregivers with poor malaria knowledge. Children whose caregivers considered artemether the best treatment for malaria were 5 times more likely to be treated with artemether (Table 4).

**Table 4:** Factors associated with receiving artemether as treatment by multiple logistic regression

	Unadjusted OR (95% CI)	Adjusted OR (95% CI)^a^
Distance to nearest health facility		
≤ 3 km		
> 3 km	0.61 (0.39–0.96)	
Wealth quintiles		
Poorest		
Second	2.52 (1.23–5.19)	
Middle	1.20 (0.55–2.63)	
Fourth	1.73 (0.82–3.61)	
Richest	2.95 (1.42–6.14)	
Symptoms		
Nausea	1.88 (1.13–3.15)	
Weakness	0.57 (0.35–0.94)	0.47 (0.28–0.78)
Caregiver age (years)		
15–24		
25–34	1.37 (0.77–2.44)	
35–44	1.07 (0.55–2.09)	
45–54	0.58 (0.27–1.25)	
≥ 55	0.23 (0.06–0.87)	
Education		
Primary School or less		
Secondary school or more	2.17 (1.39–3.41)	
Malaria knowledge		
Poor		
Good	2.41 (1.55–3.76)	1.83 (1.14–2.92)
Best treatment		
Artemether	4.90 (2.97–8.06)	5.07 (2.93–8.78)

^a^Only for the variables that stay in the model

## Discussion

The use of artemisinins, such as artemether, as the first choice treatment for malaria is a dangerous practice that could increase the emergence and spread of artemisinin resistance and reduce the possibilities of malaria control. The lack of adherence to a malaria first line treatment has been described before [24], but the treatments used were former first-line therapies, including chloroquine, amodiaquine, SP, or other artemisinin-based combinations [16, 24–26]. For the first time, wide use of artemether as the first-choice treatment against malaria is described in sub-Saharan Africa.

The *P. falciparum* resistance to artemisinin and its derivatives has spread in Southeast Asia and is threating malaria control efforts in sub-Saharan Africa. K13-propeller mutations have become the molecular marker of artemisinin-resistance detection. This mutation has been found in a Chinese migrant worker returned home from Equatorial Guinea [27], but studies has not yet detected the *k13* mutations linked with artemisinin resistance in the local population [28]. A therapeutic efficacy study is in progress in Equatorial Guinea. This study will include a molecular analysis of the samples to detect delayed parasite clearance of artemisinin. The correct interpretation of results from this study will require knowing the real pressure of artemisinins treatments in the area.

In the Bata district of Equatorial Guinea, the educational level of the caregivers, living close to a health facility, and the caregiver’s knowledge about malaria were factors associated with considering artemether the best treatment. Moreover, the caregiver’s knowledge about malaria and considering artemether the best treatment were associated with receiving artemether as treatment in the district.

The low use of ACT in Africa is usually related to the caregiver’s limited awareness about malaria [29], low socioeconomic level, and rurality [30]; although, in the Bata district, the profile of caregivers whose children received artemether was very different. Caregivers were younger than 35 years, had at least a secondary school education, lived in urban areas no further than 3 km from a health facility, and had a good Malaria Knowledge Score, a profile very similar to that of the caregivers who preferred artemether as treatment.

In the Bata district, living near a health facility seemed to be an important factor associated with considering artemether the best treatment. At the same time, considering artemether the best therapy was associated with receiving artemether as treatment. It seems that patients’ preferences were influencing the treatment received. Other studies have identified that the prescriptive practices of health workers frequently correspond with the expectations of the community, who want prompt recovery and injectable medications [17]. Furthermore, being in the area of a health care facility in the Bata district seemed to influence patients’ treatment preferences. Usually, health workers are most influential in caregivers’ decisions about the illness, when consulted [31].

Decisions about the choice of treatment are also based on perceived therapy effectiveness [17]. Artemether rapidly eliminates asexual parasite stages and early sexual forms of falciparum malaria, producing a rapid clinical and parasitological response [32, 33]. Moreover, in the Bata district, the idea that AS/AQ has adverse effects is deeply rooted in the whole population, despite the introduction of a single dose combination in 2012. Sometimes practitioners either refuse to prescribe AS/AQ to patients or they reduce the drug dosage to minimize the adverse effects [16]. Receiving artemether as treatment in the Bata district did not seem to be related to particular symptoms, which would be expected if the therapy was used according to the severity of the child, as suggested in the National Malaria Treatment Guide [10]. On the contrary, weakness was the only symptom associated with artemether, but children with weakness were 2.14 less likely to receive artemether therapy. Further research on health workers’ practices related to malaria treatment administration is needed to understand the patterns and reasons for prescribing artemether.

Treatment costs should be a key issue in deciding which antimalarial to treat with, especially among rural households [30]. However, in the Bata district, receiving artemether was more expensive than first-line AS/AQ. It seems that the high cost of artemether versus the cost of AS/AQ is not a determining factor in the preference for treating with artemether.

Caregivers who know that ACT is the government-recommended antimalarial are usually more likely to demand a first-line anti-malarial [29]. The extended preference for using artemether to treat malaria in the Bata district, regardless of where treatment was received [14], indicates it is necessary to promote the use of ACT at household and practitioner levels.

This study has some limitations. First, this is a cross sectional study conducted only in the Bata district; thus, the findings might not be generalizable to the whole country. Also, results are based on the treatment reported by caregivers and not by direct observation. However, artemether was not included as an answer in the questionnaire and was still mentioned as a preferred and received treatment more than a hundred times.

## Conclusions

This study identified the behaviours and associated factors that play important roles in the preference and use of artemether as malaria treatment. To improve the compliance with malaria first-line treatment, caregivers, public and private health staff, and drug distributers need to understand the importance of using ACT to treat uncomplicated malaria and the dangers of using artemisinin monotherapy.
